# Response of physiological characteristics of ecological restoration plants to substrate cement content under exogenous arbuscular mycorrhizal fungal inoculation

**DOI:** 10.3389/fpls.2022.1028553

**Published:** 2022-11-23

**Authors:** Qian Shu, Dong Xia, Yueyang Ma, Yang Zhang, Ting Luo, Jiaxin Ma, Fang Liu, Shuxing Yan, Daxiang Liu

**Affiliations:** ^1^ College of Biological & Pharmaceutical Sciences, China Three Gorges University, Yichang, China; ^2^ Hubei Provincial Engineering Research Center of Slope Habitat Construction Technique Using Cement-based Materials, China Three Gorges University, Key Laboratory of Mountain Hazards and Surface Processes Chinese, Yichang, China; ^3^ College of Hydraulic & Environmental Engineering, China Three Gorges University, Yichang, China; ^4^ College of Civil Engineering & Architecture, China Three Gorges University, Yichang, China

**Keywords:** vegetation concrete, arbuscular mycorrhizal fungi, photosynthetic physiology, ecological restoration, substrate cement content

## Abstract

**Introduction:**

In order to solve the inhibition of alkaline environment on plants growth at the initial stage of Eco-restoration of vegetation concrete technology, introducing AMF into vegetation concrete substrate is an effective solution.

**Methods:**

In this study, *Glomus mosseae* (GM), *Glomus intraradices* (GI) and a mixture of two AMF (MI) were used as exogenous inoculation agents. *Festuca elata* and *Cassia glauca* were selected as host plants to explore the relationship between the physiological characteristics of plants and the content of substrate cement under exogenous inoculation of AMF.

**Results:**

The experiment showed that, for *festuca elata*, the maximum mycorrhizal infection rates of inoculation with GM, MI were when the cement contents ranged 5–8% and that of GI inoculation was with the cement contents ranging 5–10%. Adversely, for *Cassia glauca*, substrate cement content had little effect on the root system with the exogenous inoculation of AMF. Compared with CK, the effects of AMF inoculation on the physiological characteristics of the two plants were different. When the cement content was the highest (10% and 8% respectively), AMF could significantly increase(*p*<0.05) the intercellular CO_2_ concentration (Ci) of *Festuca elata*. Moreover, for both plants, single inoculation was more effective than mixed inoculation. When the cement content was relatively low, the physiological characteristics of *Cassia glauca* were promoted more obviously by the inoculation of GI. At higher cement content level, inoculation of GM had a better effect on the physiological characteristics of the two plants.

**Conclusion:**

The results suggest that single inoculation of GM should be selected to promote the growth of *Festuca elata* and *Cassia glauca* in higher alkaline environment.

## 1 Highlights

- Arbuscular mycorrhizal infection of plant roots had an effect on *festuca elata* **(MI<GM<GI)**, but had no significant effect on *Cassia glauca*.
- Compared with non-inoculation, AMF inoculation could improve(p<0.05) the germination rate, root-shoot ratio (Rs) and the net photosynthetic rate (Pn) of the two plant species.
- AMF may help plants adapt to the changing alkaline soil environment, and single inoculation of AMF(GM) has the best promotion effect on plant growth and development in alkaline environment since there is a competitive relationship between the two AMFs when they are inoculated at the same time.

## 2 Introduction

Due to the rapid economic growth, the ecological environment has to some extent been destroyed and the earth’s resources and energy are becoming increasingly scarce. Therefore, people are paying more attention to the protection of ecological environment and the sustainable development of the earth (Cheng et al., 2020). The construction of transportation and water conservancy projects, in particular, has destroyed the ecological environment and produced many exposed slopes around the project sites (Wei et al., 2019). The exposed slopes are very prone to environmental problems such as debris flow and landslides (Tian et al., 2021). It is an urgent need to carry out ecological restoration of slopes. Traditional mechanical means could improve slope stability, but they are unable to meet the growing requirements of ecological environment protection and sustainable development (Li et al., 2018). So, the slope restoration technology combined with ecological means has been developed (Jing et al., 2021). Slope ecological restoration technology (He et al., 2019) is also known as green slope protection technology (Qi, 2020), vegetation slope protection engineering technology (Shi et al., 2012), and slope greening technology (Zou et al., 2019). It refers to the damaged slope stability-improving technology that combines living plant materials with non-living plant materials and civil engineering technology. Eco-restoration of vegetation concrete technology (Peikert et al., 2017; Cheng et al., 2020) makes the base material adhere to the rock surface through the effect of wire mesh, anchor and cement laid on the rock surface, creating a stable growth environment for slope vegetation. It has the dual functions of engineering protection and ecological greening and has broad application prospects in slope protection and ecological construction (Sharma et al., 2020). The technology mainly studies microbial functional diversity under different remediation methods and physical and chemical properties of soil under different climate conditions (Zhang et al., 2018; Shahzad et al., 2021). However, the use of the technology under alkaline conditions and the addition of arbuscular mycorrhizal fungi have not been studied.

AMF as common microbial remediation materials, can form mutualistic symbiosis with most terrestrial plants (Si et al., 2018; Gu et al., 2019) and are particularly common plant symbiotic bacteria in the nature. The two special structures——vesicles and jungles——can help plants and fungi to carry out good material exchange, so that the host has stronger nutrient absorption capacity (Larissa et al., 2019). Mycorrhizal consortium can promote root growth of plants, increase plant nutrient uptake, help plants resist pathogenic microorganisms (Brodersen et al., 2018), enhance the tolerance of organisms to stress, accelerate plant growth and population establishment, optimize vegetation distribution and improve ecological pattern (Śliwa et al., 2020). Studies have shown that AMF inoculation can change stomatal aperture, improve osmotic adjustment and enhance drought resistance of apple seedlings (Huang et al., 2018). Inoculation of AMF can provide effective physiological defense for plants and inhibit the invasion of multiple pathogens (Ahammed et al., 2020) and enhance plant resistance to Fusarium wilt (Li et al., 2019). Inoculation of AMF can improve maize yield, stress resistance and soil enzyme activity (Song et al., 2020). Bi has studied the effect of root damage on plant growth through indoor simulation of ground fissures in coal mining subsidence and found that inoculation of AMF can reduce the mechanical damage of roots (Bi et al., 2019). In the process of soil salinization, the transformation of effective mineral elements to insoluble salt is intensified, and a large Na ^+^ increase destroys the ion balance of plant rhizosphere, which is not conducive to plant nutrient absorption (Yang et al., 2021). In heavy metal-contaminated soil, AMF, combined with biochar, can improve the physical and chemical properties of soil, reduce the bioavailability of heavy metals in soil (Liu et al., 2018), and affect soil microbial activity and abundance and reduce direct damage of heavy metals to plants (Yuan et al., 2011). Therefore, AMF is often used as a biological fertilizer in ecological restoration.

In this study, GM, GI, and a mixture of two AMF(MI) were used as exogenous inoculation agents, *Festuca elata*and and *Cassia glauca* were taken as host plants to explore the relation between physiological characteristics of the two plants and substrate cement content under exogenous inoculation of AMF. The measurement parameters included net photosynthetic rate (Pn), transpiration rate (Tr), stomatal conductivity (Gs)and Ci, and the infection rates of plant roots were calculated.

## 3 Materials and methods

### 3.1 Experimental design

In this experiment, the AMF were all from the Institute of Plant Nutrition, Resources and Environment, Beijing Academy of Agricultura Sciences, and were used for the test after propagation. There were 48 treatments, each treatment was replicated 6 times, and all together there were 288 potted plants in the experiment.

Each pot was filled with 2000g soil. In addition, sawdust (organic matter) and habitat matrix modifier were added. The setting of cement content was based on the national industry standard *Technical Code for Eco-restoration of Vegetation Concrete on Steep Slope of Hydropower Projects*. Inactivated AMF inoculants were inoculated to ensure consistency of microflora (**Table 1**). Dosage was decided by referring to the Bank of Glomeromycota in China (BGC) (Bachir et al., 2017).

**Table 1:** Experimental base material configuration disposition.

Host plants		Cement content	Exogenous inoculation treatments of AMF(g)			
		(%)	CK	GM	GI	MI
herb	*Festuca elata*	0,5,6,7,8,10	GM:0 GI:0	GM:25 inactivated GI:25	inactivated GM:25 GI:25	GM:25 GI:25
shrub	*Cassia glauca*	0,4,5,6,7,8,				

### 3.2 Data analysis

Seed germination rate was calculated from the second day after sowing. Seed germination of *Festuca elata* was recorded every 2 days for 20 consecutive days and that of *Cassia glauca* was recorded every 3 days for 30 consecutive days. (Xu, 2012). At any stage, seed germination rate was calculated according to the ratio of germination rate(n) to total number of sowing seeds(N):

Seed germination rate (%) =n/N*100%

Plant height, leaf number and Rs were measured every 20 days and the measurement was repeated three times. Plant height was measured by ruler and leaf number was counted. When plants were mature, the underground part and the aboveground part were separated by scissors, and they were placed in 105°C oven for 30 min, and then dried at 75°C for 48 hours. The dried parts were weighed separately, and the ratios were calculated as Rs.

The Pn, Ci, Tr and Gs of plant photosynthetic indexes were measured by LI-6400 portable photosynthesis instrument. The photosynthetic physiological indexes were measured at Day 40 after sowing.

Mycorrhizal infection rate (%) was calculated with the infection grading intensity method. And the determination mycorrhizal infection rates included fixation, transparency, preparation and estimation. This method is suitable for short-term observation (3~6 weeks) (Dai et al., 2015), so in the experiment mycorrhizal infection rate was measured every 20 days and two measurements were conducted.

### 3.3 Statistical analysis

In this study, two-factor (cement gradient method and AMF inoculation method) pot experiment was used to explore the effects of exogenous inoculation of AMF on plant growth in different alkaline environments. SPSS ver.24.0 (SPSS Inc, Chicago, IL, USA) was used for statistical analysis of the data. Origin Pro 2021(1991-2020 Origin Lab Corporation) was used for figures and tables. The effects of different inoculation methods and cement contents on the physiological characteristics of the two plants were studied using the one-way ANOVA and Pearson correlation analysis. Multiple comparisons of the means were performed using the LSD test (*P*< 0.05).

## 4 Results

### 4.1 Effect of AMF on seed germination rates

**Figure 1:**
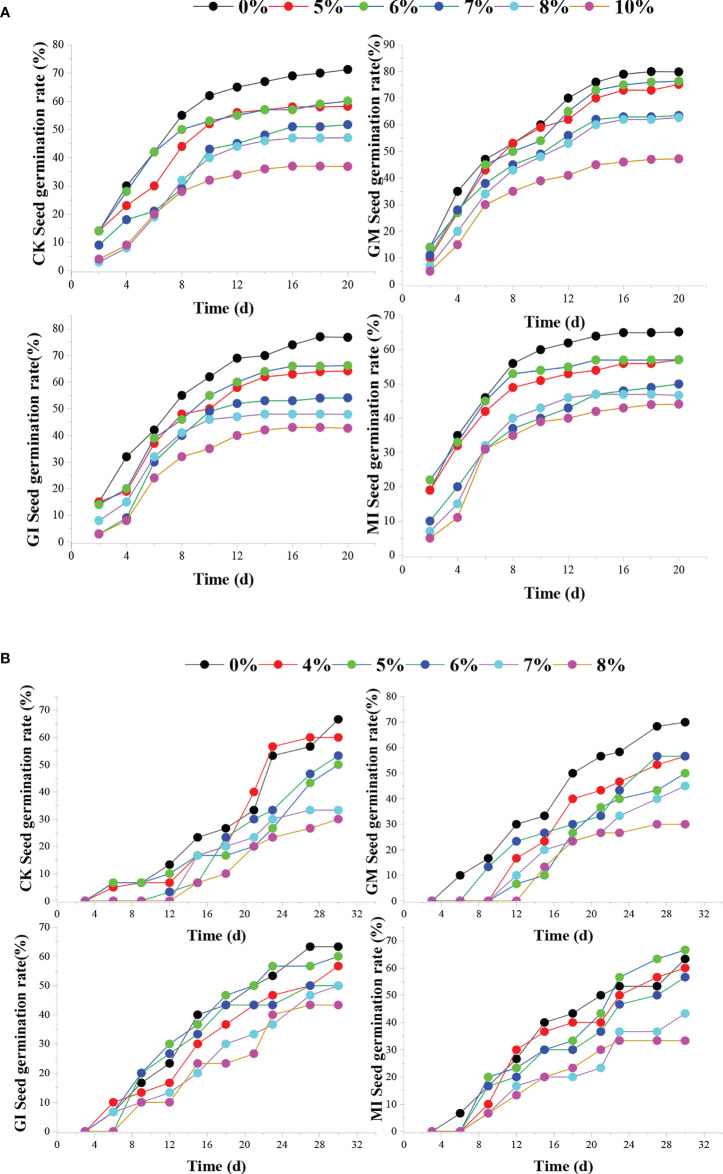
Effects of four inoculation treatments on seed germination rates of two plants under different cement contents. [(**A, B**) indicate seed germination rates of *Festuca elata* and *Cassia glauca* respectively].

**Figure 1** shows the effects of four inoculation treatments on seed germination rates of two plants under different cement contents. Without AMF inoculation (CK), the increase of cement content inhibits seed germination rate of the two plants and delays the seed germination time of *Cassia glauca*(B). Single inoculation of GM has the most obvious promoting effect on the germination of *Festuca elata* seeds. When the cement content is 5-6% and it has the most obvious promoting effect on the germination of *Cassia glauca* when the cement content is 0%. However, with the increase of cement concentration, the promotion effect of single inoculation on seed germination is weakened. In particular, the inhibition effect of single inoculation of GM is more significant. Mixed inoculation of MI has a more obvious effect on the germination of *Festuca elata* seeds at the early stage (0-6 days), and it could also shorten the germination time of the seeds of *Cassia glauca* (seedlings have emerged within 9 days). When the cement content is high, the seed germination rate of the mixed inoculation of MI is lower, which indicates that single inoculation has more obvious effect on seed germination.

### 4.2 Effects of AMF on the character of plant’s growth

#### 4.2.1 Plant height

On Day 20, compared with CK, GM inoculation and GI inoculation increase the height of *Festuca elata* by 3.96% and 5.72% respectively, and there is no significant(*p*>0.05) correlation between MI and CK. The promotion effect of GM and GI is greater than that of mixed inoculation MI. On Day 40, single inoculation of GM (0-7%) promotes the plant height of *Festuca elata* more than GI (0-6%), and mixed inoculation of MI witnesses no significant promoting effect. Compared with Day 20, the plant height of *Cassia glauca* has changed significantly. When the cement content is low or CK, AMF has no obvious effect on the plant height of *Cassia glauca*. However, with the increase of cement content, GM inoculation (4%) brings about the maximum plant height of 6.07cm. On Day 60, for *Festuca elata*, MI has a better effect on the height when the cement content is relatively low. When the cement content is 7~8%, GM inoculation and GI inoculation significantly increase the plant height (*p*<0.05). For *Cassia glauca*, when the cement content is 6%, the promotion effect of exogenous inoculation of AMF on plant height is most obvious.

#### 4.2.2 Number of blades

**Table 2** shows that the variation trend of leaf number of *Festuca elata* over time. On Day 20, low cement content (0%) is conducive to the formation of leaves (CK = 5.33). When the cement content gradually increases, the single inoculation (GM) reaches its peak, which is significantly higher (*p*<0.05) than that of other treatments, indicating that GM could promote the increase of leaf number. On Day 40, with mixed inoculation of MI, the number of leaves of *Festuca elata* increases with the increase of cement content, but GM and GI show a reverse trend, indicating that the cement content has a significant limiting effect on the single inoculation of GM and GI. On Day 60, as *Festuca elata* gradually develops and matures, the increase of leaf numbers under different cement contents decreases.

**Table 2:** Effects of four inoculation treatments on leaf number of *Festuca elata* under different cement contents.

Cement contents%	Day 20				Day 40				Day 60			
	CK	GM	GI	MI	CK	GM	GI	MI	CK	GM	GI	MI
0	5.333 ± 0.58dB	4.333 ± 0.58bB	3.667 ± 0.58bA	3.667 ± 0.54bcA	5.67 ± 0.20cA	5.333 ± 0.58cdA	5.000 ± 0.58cA	4.333 ± 0.58abB	6.000 ± 0.00cB	5.1667 ± 0.58bcA	5.1667 ± 0.20bA	4.333 ± 0.58aA
5	4.333 ± 1.53bC	5.000 ± 1.00dA	4.333 ± 0.58bB	4.333 ± 0.50cC	5.00 ± 0.58bcB	5.667 ± 0.33dcC	5.000 ± 0.67cB	4.667 ± 0.33bA	6.000 ± 0.20caB	6.333 ± 0.20cB	5.000 ± 0.56bA	5.000 ± 0.00aA
6	4.667 ± 0.58cdB	4.667 ± 0.58bB	4.000 ± 0.00bB	3.000 ± 1.00abA	5.34 ± 0.58cA	4.667 ± 0.19abB	4.667 ± 0.33abB	4.000 ± 0.33abB	5.333 ± 0.58abC	4.667 ± 0.58abB	5.000 ± 0.30abB	4.000 ± 0.10aB
7	2.667 ± 1.16abB	2.333 ± 0.58aA	3.667 ± 0.58bC	2.333 ± 0.58ab	4.67 ± 0.33abB	4.333 ± 0.33bA	4.000 ± 0.33bB	3.333 ± 0.58abA	4.667 ± 0.58bcB	4.667 ± 0.58bA	4.667 ± 0.58bB	4.333 ± 0.80aB
8	2.333 ± 0.58aA	3.000 ± 1.00ab	2.667 ± 0.58aA	2.333 ± 0.58aB	3.67 ± 0.58aA	4.000 ± 0.00bA	3.333 ± 0.20abB	3.667 ± 0.17aB	5.667 ± 0.58aA	5.333 ± 0.58bA	4.000 ± bcB0.20	4.667 ± 0.58aC
10	3.333 ± 0.58ab	2.000 ± 0.00C	2.333 ± 0.58aAB	2.333 ± 0.58aC	3.67 ± 0.58abB	2.333 ± 0.58aC	3.000 ± 0.40aA	3.333 ± 0.20aA	4.333 ± 0.58aB	4.333 ± 0.58aB	5.000 ± 0.40aA	5.333 ± 0.20aA

Based on one-way analysis of variance, lowercase letters indicate the results of different cement content treatments with the same inoculation level, and uppercase letters indicate the results of different inoculation treatments at the same cement content, and the same letter shows no significant difference (p < 0.05, The same below).

**Table 3** depicts the variation trend of leaf number of *Cassia glauca* over time.

**Table 3:** Effects of four inoculation treatments on leaf number of *Cassia glauca* under different cement contents.

Cement	Day 40				Day 60			
contents(%)	CK	GM	GI	MI	CK	GM	GI	MI
0	8.000 ± 0.20cA	10.667 ± 0.23bA	6.000 ± 0.20bA	8.000 ± 0.34bA	12.000 ± 0.46cA	7.333 ± 0.15aA	8.000 ± 0.00aB	8.000 ± 0.20aA
4	5.333 ± 1.15bA	5.000 ± 1.00aC	4.000 ± 0.20abB	3.333 ± 0.16aA	10.000 ± 0.20bcB	8.667 ± 0.15aA	7.333 ± 0.23aA	6.667 ± 0.15aB
5	5.333 ± 1.15bB	4.667 ± 0.23aA	4.667 ± 0.23abB	3.333 ± 0.23aA	6.667 ± 0.15abB	6.000 ± 0.00aB	4.667 ± 0.23aC	6.667 ± 0.15aA
6	6.667 ± 1.15bcB	3.333 ± 0.23aB	6.000 ± 0.00bB	7.333 ± 0.23bA	7.333 ± 0.15abB	8.667 ± 0.20aB	7.333 ± 0.10aC	8.000 ± 0.10aA
7	2.667 ± 1.15aB	3.333 ± 1.15aB	6.667 ± 0.30bA	2.667 ± 0.15aAB	5.333 ± 0.15aAB	8.667 ± 0.15aB	6.000 ± 0.20aA	7.333 ± 0.15aAB
8	2.000 ± 0.00bB	3.333 ± 0.23aAB	2.000 ± 0.00aB	2.667 ± 0.15aA	6.000 ± 0.20aB	6.000 ± 0.00aB	7.333 ± 0.16bA	5.333 ± 0.15aA

On Day 20, the number of leaves is 2, the pair of cotyledons of leguminous plants when they emerge. With the extension of time, the leaves increase (*p*<0.05) significantly. When the cement content is 0%, exogenous inoculation of GM is conducive to the increase of the leaves. Compared with mixed inoculation, single inoculation of AMF demonstrates greater promoting effect.

#### 4.2.3. Rs

For *Festuca elata* (**Figure 3**), when the cement content is 0%, CK shows the maximum of Rs (76.38%) and GI shows the minimum 66.62%. With the increase of cement content, Rs of AMF inoculation is higher than that of CK. When the cement content is the highest (10%), Rs of single inoculation of GM is the highest (74.24%), which is 13.05% higher than that of CK, indicating that GM has a significant promoting(*p*<0.05) effect.

Compared with *Festuca elata*, the Rs of *Cassia glauca* is generally lower (less than 50%), and the 51.93% of the single inoculation of GM (8%) is an exception. Different from *Festuca elata*, when the cement content is 0%, AMF inoculation could significantly increase (*p*<0.05) the Rs. With the increase of cement content, GI and MI have advantages in promoting Rs. However, when the cement content is the highest (8%), GM inoculation demonstrates greater promoting effect than CK inoculation, but there is no significant difference(*p*>0.05) between the two.

### 4.3 Effects of AMF on plant photosynthesis

The Pn of the two plants under different cement concentrations is shows in **Figures 4a, A**. For *Festuca elata*, under different cement contents, the inoculation of AMF can promote the Pn of plants. When the cement content is 0%, the Pn of the mixed inoculation MI is 1.62% higher than that of CK. With the increase of cement content, single inoculation has more advantages than mixed inoculation. When the cement content is 6%, the Pn of GM is 10.60% higher than that of CK. When the cement content is 7%, the Pn of GM and the Pn of GI increase 3.16% and 9.58% respectively compared with CK, but there is no significant difference between GM and CK (*p*>0.05). For *Cassia glauca*, there is a positive correlation between net photosynthetic rate and cement content (0-6%) when it is not inoculated with AMF. The Pn of the plants inoculated with MI is less than 7μ mol·m^-2^·s^-1^, which indicates that MI inoculation reduces the Pn. Only when the cement content is 0~4% can the Pn of *Cassia glauca* be increased by GM inoculation.

The Gs of the two plants under different cement concentrations is shows in **Figures 4b, B**. For *Festuca elata*, when AMF is not inoculated, the Gs increases with the increase of cement content under the condition of low cement content (0-6%), while under the condition of high cement content, the opposite trend appears. After the inoculation with AMF, the Gs of plants reaches the peak (5%). Compared with CK inoculation, GM and GI increase by 39.91% and 16.36% respectively, and MI decreases by 3.67%. For *Cassia glauca*, when AMF is not inoculated, the Gs decreases with the increase of cement content. When GM inoculation is performed, the Gs is positively correlated with the cement content. In the condition of high cement, the three inoculation methods could all improve the Gs, but the promoting effect of GI inoculation is more obvious (more than 2μ mol·m^-2^·s^-1^).

The Ci of the two plants under different cement concentrations is shows in **Figures 4c, C**. For *Festuca elata*, when the cement content is 0%, the Ci concentration is the highest with inoculation of MI, which is 1.44% higher than that of CK. When the cement content is 8~10%, the second peak of Ci appear, with Ci of MI being the highest. This indicates that mixed inoculation has the most obvious effect on Ci. For *Cassia glauca*, When the cement content is 0~4%, GM and GI can increase Ci. At a higher cement content level, AMF inoculation can increase Ci, but the promoting effect of single inoculation is better than that of mixed inoculation, and GI presents the most obvious promoting effect.

The Tr of the two plants under different cement concentrations is shows in **Figures 4d, D**. Without cement, GI has the lowest Tr. When cement content is 5%, AMF inoculation could improve Tr of leaves. When the cement content is 6-7%, the Tr of GI and MI are lower than that of CK. When the cement content is 8%, Tr of CK is the lowest (15.40mmol·m^-2^·s^-1^). When the cement content is the highest (10%), AMF inoculation inhibits the Tr of plants. For *Cassia glauca*, the Tr of plants inoculated with GM is the highest under low cement conditions, and the Tr of plants inoculated with GI and MI are 51.61% and 66.92% higher than that of CK under the condition of high (7%) cement content.

### 4.4 Plant mycorrhizal infection rates

On Day 20, for *Festuca elata*, the inoculation of GI has a worse effect on the root infection of plants, while the inoculation of MI has a better effect.

On Day 40, for *Festuca elata*, the change trends of mycorrhizal infection rate in GM inoculation and MI inoculation are similar to those on Day 20. But the difference is that when the peak value of mycorrhizal infection appears, the cement content is 7%, while on Day 20, the cement content is lower (*p*<7%). For *Cassia glauca*, Compared with *Festuca elata*, external inoculation of AMF has less effect on *Cassia glauca* at Day 40 (**Figure 5c**). Changes in cement content do not affect plant root infection of AMF. When the cement content is relatively low, the mycorrhizal infection rate of *Cassia glauca* inoculated with MI is always higher than that of single inoculation. But when the cement content increases to 7%, the experimental results are the opposite.

### 4.5 Correlation analysis between cement contents and growth characteristics of two plant species

Correlation analysis of different inoculation methods, cement contents and growth characteristics of *Festuca elata* is as shown in **Table 4**. The result shows there is a significantly positive correlation (*p*<0.01) between cement content and Ci.

**Table 4:** Correlation analysis of different inoculation methods, cement content and growth status of *Festuca elata*.

	Seed germination rate	H20	H40	H60	N20	N40	N60	Rs	Pn	Gs	Ci	Tr
**cement content**	-0.834**	-0.851*	-0.936**	-0.938**	0.671**	-0.661**	-0.444**	-0.360*	-0.513**	-0.538**	0.420**	-0.260
**Seed germination rate**		0.772**	0.843	0.792*	0.603*	0.655**	0.440*	0.248	0.494**	0.566**	-0.231	0.277
**H20**			0.854*	0.833*	0.599	0.536	0.262	0.311*	0.609*	0.532**	-0.194	0.318*
**H40**				0.939**	0.620	0.626*	0.352*	0.383**	0.532*	0.491**	-0.369**	0.301*
**H60**					0.614**	0.605*	0.406**	0.377**	0.496**	0.574*	-0.417**	0.349*
**N20**						0.673*	0.435**	0.275	0.431**	0.612**	-0.470**	0.349*
**N40**							0.673**	0.240	0.342*	0.539**	-0.425**	0.405**
**N60**								0.408**	0.042	0.514**	-0.327*	0.295*
**Rs**									0.012	0.090	-0.163	-0.031
**Pn**										0.409**	0.042	0.350*
**Gs**											-0.427**	0.606**
**Ci**												-0.278

Abbreviations: Rs is root-shoot ratio (%), Pn is net photosynthetic rate (μ mol·m-2·s-1), Gs is stomatal conductivity (μ mol·m-2·s-1), Ci is intercellular CO2 concentration (μ mol·mol-1), and Tr is transpiration rate (m mol·m-2·s-1), H is plant height, N is number of blades. * means a significant indigenous correlation (p<0.05), ** means a very significant indigenous correlation (p<0.01). Positive value indicates positive correlation, and negative value indicates negative correlation.

And the cement content is negatively correlated (*p*<0.01) to germination rate, Day 40 plant height, Day 60 plant height, Pn and Gs.

Correlation analysis of different inoculation methods, cement contents and growth characteristics of *Cassia glauca* is as shown in **Table 5**.There are significant positive correlations (*p*<0.01) among germination rate and plant height and leaf number, between Pn and Tr、Gs, between Ci and Tr. Cement content is extremely negatively correlated (p< 0.01) to germination rate, plant height, leaf number(Day 40and Day 60), Gs, Day 40 plant height and Rs.

## 5 Discussion

### 5.1 Response of plant growth to different cement contents under exogenous AMF inoculation

In this study, the effects of AMF on plant growth are measured by the germination rate, plant height, leaf number and Rs under the conditions of different cement contents.

The research has found that higher cement content has inhibited seed germination, plant height, leaf number and Rs without AMF inoculation, because the addition of cement increases soil Ph (Li et al., 2021), which is not conducive to nutrient absorption of plants (Glenda et al., 2019).

**Figure 2:**
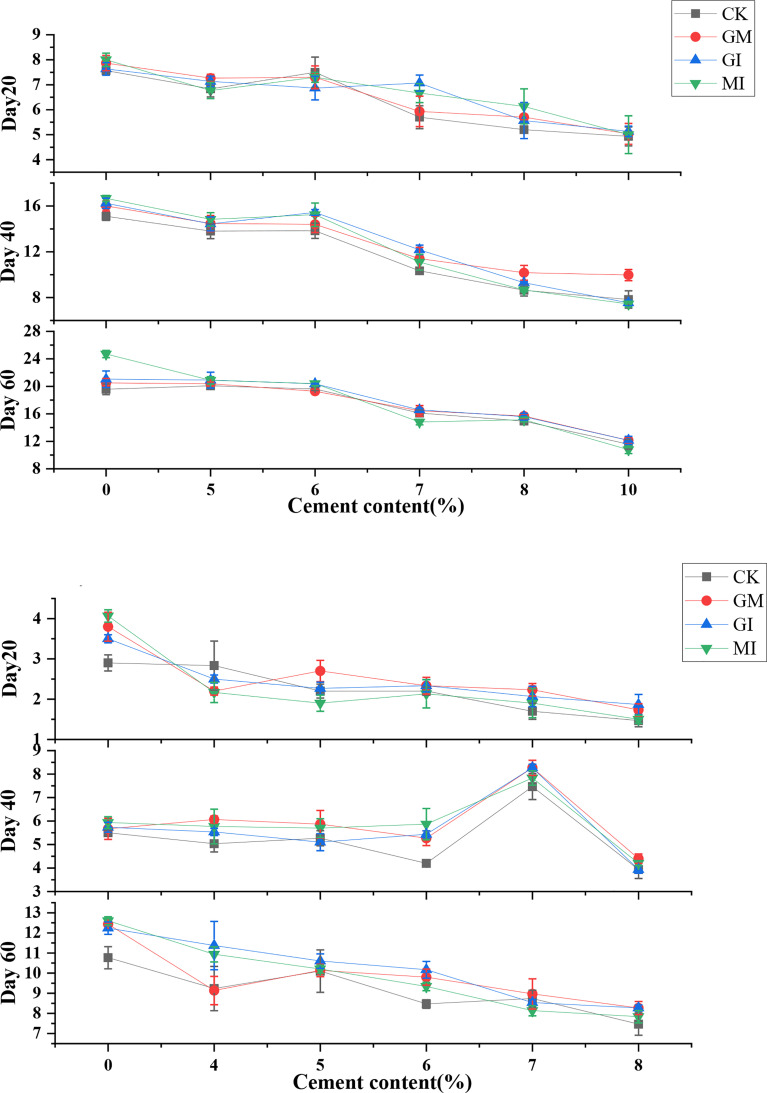
Effects of AMF on plant height of two plant species under different cement contents. (e and E represent *Festuca elata* and *Cassia glauca* respectively).

For *Festuca elata*, the germination rate of seeds with GM inoculation is the highest, in particular, the cement content is 5% and 6%, this is because mild alkaline soil is conducive to GM mycorrhizal infection and mycelial extension (Sun et al., 2020; Huang et al., 2020), The overall effect of GI (**Figures 1A, B**) inoculation is not obvious, especially when the cement content is relatively high, and this might be related to the preferred soil pH range of the two AMFs (Dominika et al., 2017).For *Cassia glauca* (**Figure 1B**), inoculation of GI and MI promoted the number of seed germination and advanced the germination time (Josiane et al., 2019; Giupponi and Leoni, 2020; Mohammad and Hassan, 2020). Moreover, the germination rate of the two plant species has an extremely significant negative (*p*< 0.01) correlation with cement content (**Tables 4**, **5**). **Figure 2** shows that with the increase of cement content, the plant height of two plants have a downward trend. At early stage, compared with CK, AMF infection can improve plant nutrient absorption capacity (Zhang, 2017), help biomass accumulation of the aboveground part of plant (Chmura et al., 2021), resulting in seedling elongation. In the medium term, the promotion effect of MI inoculation is better at low cement content level (0-5%). However, for both plants, when the cement content is relatively high, the promotion effect of single inoculation of AMF is more obvious. This is because the nutrient availability is reduced in alkaline environment (Ashley et al., 2019), leading to competition between the two AMFs (White, 2019; Caroline et al., 2020; Wang et al., 2021).

**Table 5:** Correlation analysis of different inoculation methods, cement content and growth status of *Cassia glauca*.

	Seed germination rate	H20	H40	H60	N40	N60	Rs	Pn	Gs	Ci	Tr
**cement content**	-0.879**	-0.769**	-0.729**	-0.839**	-0.531**	-0.327**	0.256	0.082	-0.108**	-0.079	-0.035
**Seed germination rate**		0.682**	0.672**	0.764*	0.504**	0.418**	-0.277	-0.032	0.083	0.097	0.038
**H20**			0.559**	0.809**	0.591**	0.173	-0.074	-0.196	-0.051	0.033	-0.130
**H40**				0.652**	0.389**	0.267	-0.441**	-0.006	0.126*	0.049	0.068
**H60**					0.487**	0.158	0.010	-0.213	-0.113	-0.011	-0.186
**N40**						0.245	-0.022	0.090*	-0.037	0.151	-0.117
**N60**							0.129	0.265*	0.114	0.028	0.178
**Rs**								-0.041	0.321*	0.010	-0.341*
**Pn**									0.312*	-0.159	0.431**
**Gs**										0.595	0.814**
**Ci**											0.557**

Abbreviations: Rs is root-shoot ratio (%), Pn is net photosynthetic rate (µ mol·m-^2^·s-^1^), Gs is stomatal conductivity (µ mol·m-^2^·s-^1^), Ci is intercellular CO2 concentration (µ mol·mol-^1^), and Tr is transpiration rate (m mol·m-^2^·s-^1^), H is plant height, N is number of blades. * means a significant indigenous correlation (p< 0.05), ** means a very significant indigenous correlation (p<0.01). Positive value indicates positive correlation, and negative value indicates negative correlation.

**Figure 3:**
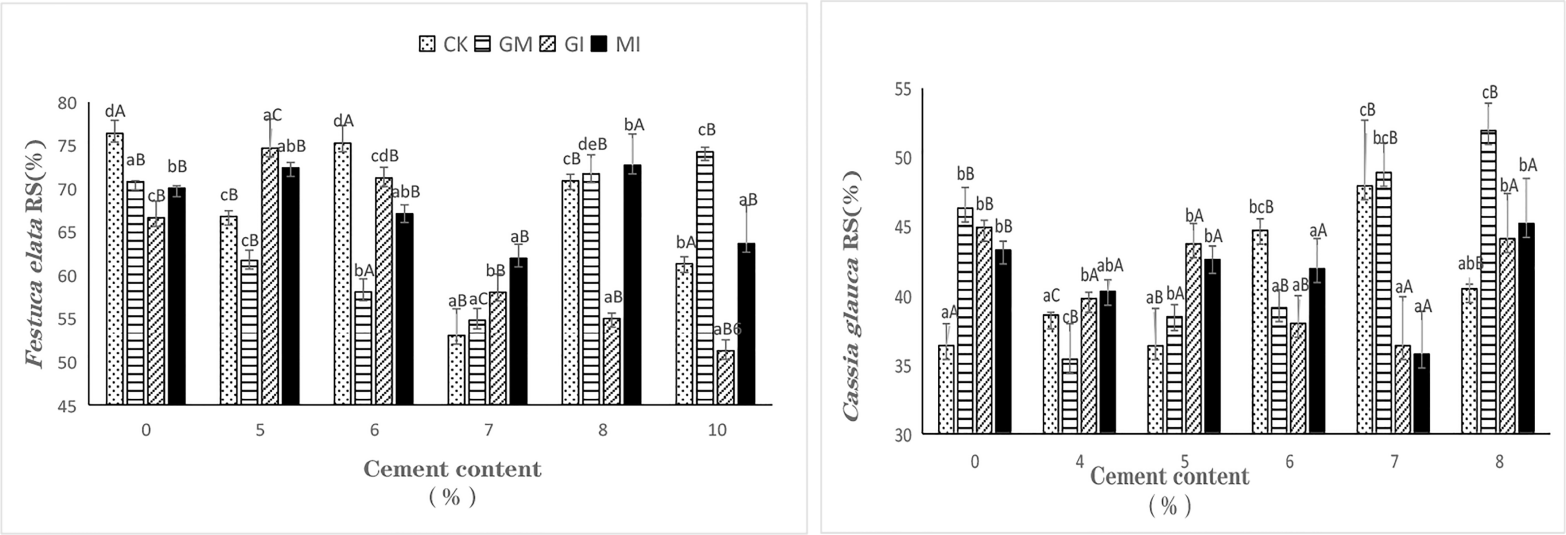
Effects of four inoculation treatments on Rs of two plants under different cement contents. Based on one-way analysis of variance, lowercase letters indicate the results of different cement content treatments with the same inoculation level, and uppercase letters indicate the results of different inoculation treatments at the same cement content, and the same letter shows no significant difference (p<0.05).

For *Festuca elata*, the number of leaves has a significant negative correlation with the inoculation method and cement content (p<0.05) (**Table 2**). Therefore, AMF inoculation has no effect on the number of leaves. But some researchers have shown that inoculation of AMF can help carbohydrate transfer and accumulation, and improve plant photosynthetic efficiency by increasing the leaf area of symbiotic plants (Cao et al., 2020). Therefore, it can be speculated that the biomass allocation of AMF is to increase the leaf area rather than the number of leaves. **Figure 3** shows the variation of Rs with cement content in two plants inoculated with AMF. The increase of Rs is a way to improve stress resistance of plants (Birnbaum et al., 2018). When in the environment of high cement content, the inoculation of GM can alleviate the sensitivity of plants roots to stress, changing the distribution mode of aboveground and underground parts and improving their stress resistance (Eva et al., 2020; Azizi et al., 2021). In particular, *Cassia glauca* is a legume, and AMF can synergistically promote its rhizobia, thereby significantly increase the biomass of aboveground and underground parts of plant (*p*< 0.05), thus promoting the number of leaves and the Rs (Li et al., 2021).

### 5.2 Response of plant photosynthesis to different cement contents under AMF inoculation

**Figure 4:**
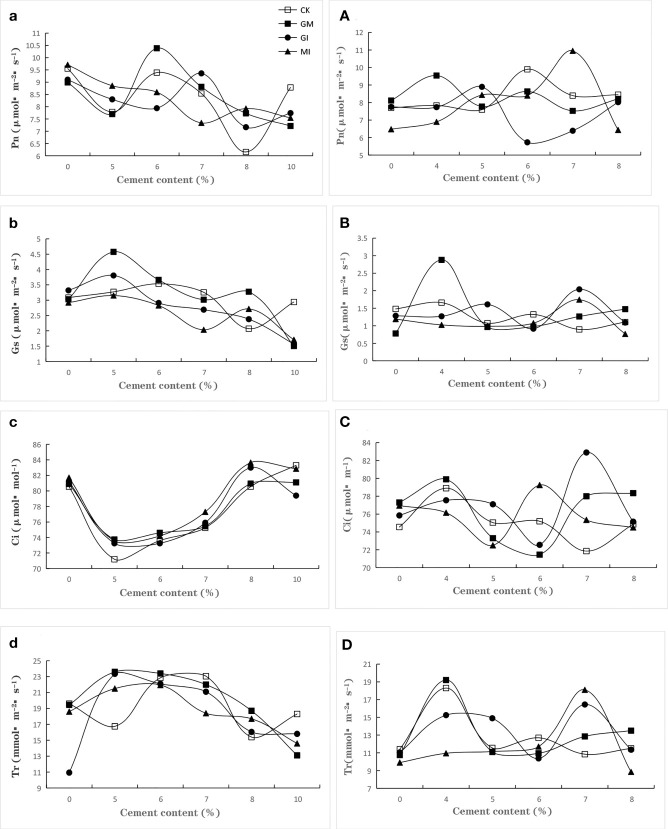
Effects of four inoculation treatments on plant photosynthesis under different cement contents. (**a-d**: *Festuca elata;* **A-D**: *Cassia glauca*, respectively).

Figure4 shows the response of photosynthesis of host plants to cement content under exogenous inoculation with AMF. This experiment has measured four (Pn, Gs, Ci and Tr) indexes respectively.

The experimental results show that after adding cement, inoculation of AMF could improve the Pn and Ci of the two plants. For *Festuca elata*, the higher the cement content is, the greater effect the inoculation of AMF has on the Pn (**Figure 4a**) and Ci (**Figure 4c**). When cement content is lower, MI inoculation can significantly increase (*p*<0.05) Ci. This is because after AMF infects plant roots, the Pn and Ci of plants can be improved by increasing the contents of chlorophyll a and chlorophyll b (Ali et al., 2019) and increasing the surface area of single leaf (Zhu et al., 2008), and the mycelial bridge can also improve the accumulation of carbohydrates by improving the photosynthetic fluorescence characteristics of plants (Azizi et al., 2021; Bellido et al., 2021). For *Cassia glauca*, AMF inoculation can improve Pn (**Figure 4**) and Ci (**Figure 4**), but the promotion effect of single inoculation is better than that of mixed inoculation, especially the inoculation of GI. But when the cement content is relatively high, MI is more dominant. The reason is that the products secreted by two AMFs during competitive infection affect the substances related to photosynthetic regulation in plants growth and development (White, 2019; Caroline et al., 2020).

**Figure 5:**
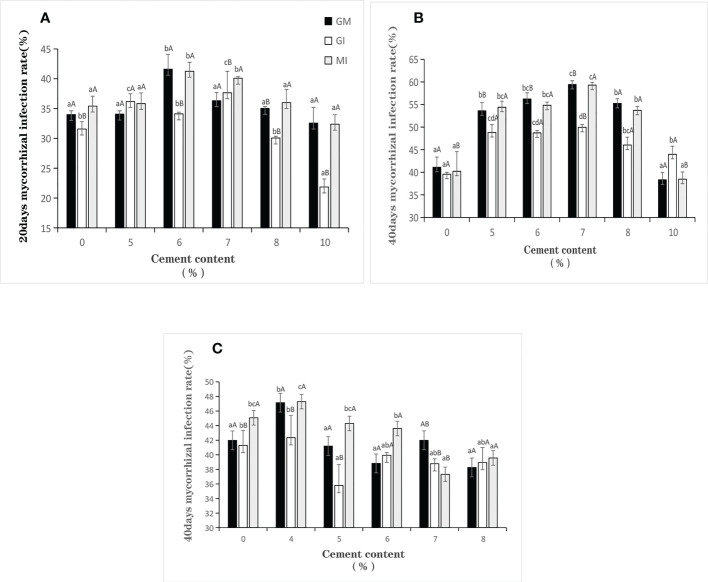
Mycorrhiza infection rates of two plants under different cement contents. [**(A, B)** infection rates of Festuca elata at Day 20 to Day 40; **(C)** infection rate of Cassia glauca at Day 40]. Based on one-way analysis of variance, lowercase letters indicate the results of different cement content treatments with the same inoculation level, and uppercase letters indicate the results of different inoculation treatments at the same cement content, and the same letter shows no significant difference (p<0.05).

The Gs of *Festuca elata* (**Figure 5b**) is not significantly affected by the inoculation of AMF under the conditions of different cement content levels, which may be due to the fact that under the stress of cement, *Festuca elata* obtain more CO_2_ (Dan et al., 2016; Bao et al., 2020) with the least water loss by reducing stomatal opening (Wang et al., 2021). But the Gs of *Cassia glauca* (**Figure 5B**) increases, and there was a positive correlation between Gs and the cement content with 8% cement content being an exception. As mentioned above, after the inoculation of AMF, the root system of plant is affected by the combined action of AMF and rhizobia (Sonnemann et al., 2012), which improves the root water absorption efficiency (Li et al., 2021), so the stomatal closure is less.

**Figure 4d** proves that the increase of cement content affects the development of *Festuca elata* leaves and reduces the number of stomata. When the cement content is 10%, Tr of the AMF inoculation is lower than that of CK inoculation, which is consistent with that of most researchers (Zhang et al., 2018; Michael et al., 2018; Mohammad et al., 2019). The researchers believe that AMF regulates water use efficiency in plants by reducing transpiration rate to improve plant photosynthetic efficiency. The effect of AMF *Cassia glauca* on Tr is not obvious (**Figure 4**), which might be because the leaves are small and the measured transpiration rate is not representative.

### 5.3 Relationship between plant root infection rates and cement contents

For *Festuca elata* (**Figure 5A**), when the cement content is between 5-8%, GM and MI are more likely to infect the root system of plants. However, when the cement content is the highest, the infection rate of single inoculation of GI is the maximum, which indicates that the mycorrhizal infection rate of the inoculation of MI is mainly affected by the components of GM inoculants, which further indicates that GM has greater infection competitive advantage than GI for *Festuca elata* (White, 2019; Caroline et al., 2020).

It has been found that the effect of cement content on fungal infection of *Cassia glauca* roots is not obvious (**Figure 5**). The reasons are: *Cassia glauca* germinates and develops late; as a legume shrub, its special root system, it has the judgment of nodule affect experimental results (Ai et al., 2016; Huo et al., 2021); and errors have occurred in sampling.

## 6 Conclusions

This study has comprehensively evaluated the response of plant physiological characteristics to different cement contents under external inoculation of AMF. The experimental results show that the cement content of the substrate inhibits the growth of plants and affects their physiological characteristics. Exogenous inoculation of AMF can promote plants growth, increase plants Pn and Ci and reduce Tr of *Cassia glauca* and Gs of *Festuca elata*. And single inoculation of GM has the best promotion effect on plants growth and development in alkaline environment since there is a competitive relationship between the two AMFs when they are inoculated at the same time. In addition, the infection rate of mixed inoculation of MI shows that GM has more competitive advantages than GI for *Festuca elata*. To sum up, we suggest that to improve the growth and photosynthesis of *Festuca elata* and *Cassia glauca* in vegetation concrete (cement content is 6–8%), the scheme of single inoculation of GM should be selected.

## Data availability statement

The original contributions presented in the study are included in the article/**Supplementary Material**. Further inquiries can be directed to the corresponding author.

## Author contributions

DX: Conceptualization, Investigation, Writing - original draft. QS: Investigation, Formal analysis, Visualization. YM: Investigation, Data curation. TL: Validation, Writing - review. FL and JM: Data curation, Visualization. SY: Data curation. DL: Supervision. All authors contributed to the article and approved the submitted version.

## Acknowledgments

This research is supported by the open fund of Key Laboratory of Urban Land Resources Monitoring and Simulation, Ministry of Natural Resources (KF-2019-04-071).and the National Natural Science Foundation of China (No.51979147). The team I work with is academically enterprising and Miss Yan Yujie, in particular, has given me valuable help.

## Conflict of interest

The authors declare that the research was conducted in the absence of any commercial or financial relationships that could be construed as a potential conflict of interest.

## Publisher’s note

All claims expressed in this article are solely those of the authors and do not necessarily represent those of their affiliated organizations, or those of the publisher, the editors and the reviewers. Any product that may be evaluated in this article, or claim that may be made by its manufacturer, is not guaranteed or endorsed by the publisher.
