# Supplementary figures and images for: Response of physiological characteristics of ecological restoration plants to substrate cement content under exogenous arbuscular mycorrhizal fungal inoculation
Source: Front Plant Sci. 2022 Nov 23;13:1028553. doi: 10.3389/fpls.2022.1028553 (PMC9728102; doi:10.3389/fpls.2022.1028553)

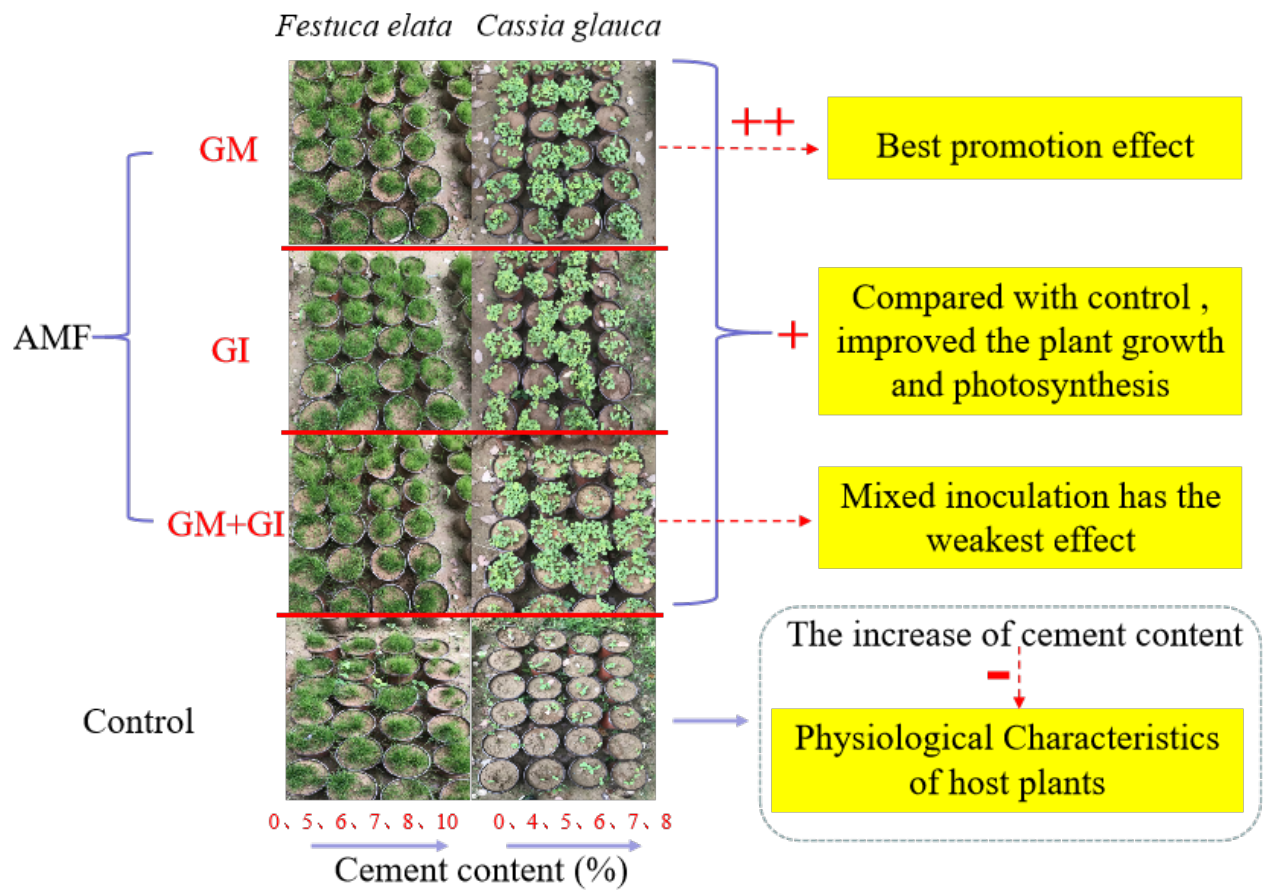

Supplement: Supplementary file 1 [file Image_1.pdf]
